# Etiology and Diagnosis for Idiopathic Condylar Resorption in Growing Adolescents

**DOI:** 10.3390/jcm12206607

**Published:** 2023-10-19

**Authors:** Eiji Tanaka

**Affiliations:** Department of Orthodontics and Dentofacial Orthopedics, Tokushima University Graduate School of Biomedical Sciences, Tokushima 770-8504, Japan; etanaka@tokushima-u.ac.jp; Tel.: +81-88-633-7356

## 1. Introduction

This article has been written in honor of the late professor emeritus Kazuo Tanne, who passed away on 4 March 2023. During his career as dean of and professor at the Hiroshima University Graduate School of Biomedical Sciences, he greatly influenced and contributed to my now comprehensive understanding of the mechanism behind mandibular condylar resorption and deformation. Moreover, for researchers around the world, he was a great inspiration with great perseverance. I hereby express my gratitude for his efforts and pray that his soul will rest in peace.

During growth, the long bones increase in length via epiphyseal cartilaginous growth and in size via periosteal growth due to new bone apposition via subperiosteal and subendoseum osteoblasts. The mandibular condylar cartilage is the primary growth center of the mandible, and bone formation in the mandibular condyle causes the mandibular rami to grow upward and backward, moving the entire mandible in the opposite directions, namely, downward and forward. This also results in an increase in the intercondylar width corresponding to the lateral growth of the cranial base [1]. Thus, the mandible, including the mandibular condyle, is subject to an intense mechanical load during growth, increases in size both vertically and horizontally, and maintains its size after growth is completed due to functional bone remodeling [2].

Idiopathic condylar resorption (ICR), on the other hand, is defined as a condition in which the mandibular condyle is specifically and progressively resorbed, accompanied by a marked reduction in mandibular ramus height; this reduction is affected by mandibular retraction and, subsequently, an anterior open bite, resulting in an imbalance between the occlusal and musculoskeletal systems [3]. Since ICR is reported to occur most commonly in teenage girls, growing ICR patients are likely to be diagnosed as suffering from a simple maxillary protrusion or mandibular retrusion and thus receive inappropriate orthodontic treatment, which may induce an exacerbation of mandibular condylar resorption [3,4,5]. Therefore, it is extremely important to diagnose ICR as early and accurately as possible; however, the diagnostic criteria for ICR are still unclear.

The purpose of this article is to present the risk factors associated with the development of ICR based on previous studies and propose criteria for the diagnosis of ICR and the timing of treatment initiation.

## 2. Etiology of ICR

The local factors of ICR include TMJ osteoarthritis, ischemic necrosis, infection, and trauma to the head, neck, and jaws [3,4,5]. The systemic factors reported include autoimmune diseases, such as rheumatoid arthritis and Sjögren’s syndrome, and female hormonal disorders [3,4,5]. In addition, ICR commonly occurs in association with orthodontic treatments and orthognathic surgery involving mandibular advancement [3].

In our clinico-statistical survey of orthodontic patients under 25 years of age who visited the orthodontic clinic of Tokushima University Hospital, a total of 12 patients with ICR were identified among 1735 participants with various malocclusions; the prevalence rate was 0.7%, and the male-to-female ratio was 1:5 [6]. The average age at onset was 12 years. All 12 patients exhibited maxillary protrusion with a retrognathic chin and an anterior open bite; however, 2 of the 12 ICR patients presented skeletal Class III malocclusion (anterior crossbite) before the onset of ICR. Four patients had undergone previous orthodontic treatment, and one had a history of oral contraceptive use. Interestingly, 75% of the ICR patients had a history of temporomandibular disorders (TMDs), and 66.7% had postural and parafunctional habits. Considering these results, the following risk factors associated with the development of ICR were identified.

### 2.1. Sex and Age

ICR appears most commonly in women in their teens or early twenties, with a sex ratio of 1:9 to 1:16 [7]. Gunson et al. [8] reported that a decreased blood concentration of 17β-estradiol is a major cause of ICR. 17β-estradiol is one of the major components of the female hormone estrogen, which is strongly associated with pregnancy and fertilization, and its decrease leads to menstrual irregularity and amenorrhea. Milam et al. [9] reported that estrogen receptors (ERβ) are abundant in female TMJs and that these receptors themselves may precipitate condylar resorption and deformity, including ICR. Estrogen secretion deficiency in premenstrual women may have a strong influence on whether they develop of ICR.

### 2.2. Mechanical Stress

Like other synovial joints in the human body, the TMJ is a widely recognized load-bearing structure, and the application of a mechanical load to the mandibular condyle is essential for its growth, development, and maintenance [10]. A healthy TMJ, for example, can resist or adapt to somewhat large loads caused by nocturnal clenching or by the use of orthodontic devices such as intermaxillary elastics, Herbst appliances, chin caps, and so on. However, if the host’s adaptive capacity decreases due to systemic and local problems, mechanical loading accelerates the destructive processes in the mandibular condyle and attenuates the defensive function of the TMJ, resulting in condylar resorption. The following factors are associated with the mechanical loading of the mandibular condyle.

#### 2.2.1. Previous Orthodontic Treatment Including Surgical Orthodontic Treatment

Abnormal or excessive mechanical loading of the mandibular condyle during orthodontic treatment is thought to be one of the risk factors contributing to the development of ICR. Although the relationship between ICR and orthodontic treatment is still unknown, it should not be ignored that many patients with ICR have undergone orthodontic treatment, even though the occurrence of ICR is extremely rare [3]. For example, in the orthognathic surgery for skeletal Class II open bite, advancing the mandible induces the anterior displacement of the distal bone segment, by which a backward retraction force caused by suprahyoid muscles is produced on the proximal bone segment, including the mandibular condyle, leading to a rapid increase in excessive compressive force on the mandibular condyle. In most cases, the articular cartilage adapts through tissue remodeling; however, in rare cases, condylar resorption occurs because the remodeling function cannot adequately combat the destructive process [3]. In particular, it has been reported that mandibular condylar resorption occurs in 67% of patients with mandibular advancement equal to 10 mm or more [11]. For patients with previous ICR, inactive condylar resorption can be reactivated by excessive stress on the mandibular condyles induced by orthognathic surgery, causing the mandible to advance more than 5 mm [12].

#### 2.2.2. History of Maxillofacial Trauma

Patients with ICR often have a history of maxillofacial trauma, especially forms related to the TMJ. Direct or indirect trauma of the mandibular condyle is thought to cause a partial loss of mandibular cartilage through laceration, resulting in bony condylar lysis of the mandible due to decreased nutritional supply and weakened tissue resistance [13,14]. Reduced mechanical loading and blood flow blockage after a mandibular condylar fracture may also lead to condylar resorption. Arnett et al. [15] demonstrated that third-molar extraction and maxillofacial trauma can generate significant loads on the mandibular condyle and precipitate surgical mandibular advancement of more than 5 mm [16]. We studied the possibility of condylar resorption in porcine mandibles using a model of mandibular condylar trauma by dropping a 200 gw impactor from a height of 60 cm onto the top of a porcine mandibular condyle and performed a morphological evaluation using micro-CT and an immunohistochemical evaluation using tissue sections [17]. The results showed that MMP-1 and IL-1β were highly expressed at the boundary between the cartilage and the subchondral bone, although no significant changes were observed in the superficial layer of the mandibular condylar cartilage after trauma.

#### 2.2.3. Abnormal Postural and Parafunctional Habits

Among children of elementary to junior high school age, the biomechanical environment in the tissues surrounding the TMJ dramatically changes in size with skeletal growth. Thus, even if the TMJs are subjected to relatively large stress during the growth period, children are relatively adaptable to environmental changes and often remain asymptomatic. Parafunctional habits and bad posture have been identified as risk factors for detrimental effects on the TMDs [18]. For example, the habit of always sleeping facing the same direction or sleeping on one’s stomach may induce abnormal and/or excessive stress on the TMJ, which may lead to TMJ damage [3,4,5]. The habit of resting with one’s chin on one’s hands induces sustained stress on the unilateral TMJ, which is thought to be more likely to damage the TMJ. It is thought that mandibular condylar resorption and deformity may develop when tissue resistance decreases, in addition to via the parafunctional and postural habits in one’s lifestyle, leading to ICR.

#### 2.2.4. Temporomandibular Joint Disc Disorders

TMD symptoms such as joint sounds, pain, and trismus are commonly presented by patients with ICR. TMJ disc disorders are also the most common signs of TMDs, and some are developed through bony changes in the mandibular condyle, while others develop without bony changes [4,13,15]. This indicates that there is no, or at least a very limited, causal relationship between disc displacement and bone changes [4]. In growing patients, TMJ disc disorders have an inhibitory effect on mandibular condylar growth [13,15]. However, patients with a growth-deficient mandible do not always have TMJ disc disorders [13,15]. Joint pain is regarded as one of the symptoms of TMD, but patients with ICR commonly have no symptoms, including joint pain [4]. On the other hand, skeletal Class II patients with active TMJ disease—such as anterior disc displacement without reduction, treated only with mandibular advancement surgery—often have poor outcomes and significant relapses due to severe condylar resorption [16]. Furthermore, degenerative and osteolytic changes make the TMJ components highly susceptible to failure under the new functional loading resulting from the orthognathic surgical repositioning of the maxillofacial skeleton [17]. Taken together, TMJ disc disorders might be a risk factor for ICR.

## 3. Diagnostic Criteria for ICR

In 2009, the Japan Intractable Diseases Information Center declared ICR to be an intractable disease, and little new information is available on the diagnostic criteria for ICR. The factors of the development of ICR are also unknown, and ICR patients are likely to visit dentists and orthopedic surgeons after an anterior open bite emerges due to a reduction in mandibular ramus height and mandibular retraction caused by progressive condylar resorption. Therefore, it is highly likely that important symptoms or signs that may be risk factors for ICR are hidden in these pathogenic factors.

As mentioned above, osteogenesis in the growing condyle causes the mandibular ramus to grow upward and backward, resulting in the entire mandible moving forward and downward [2]. Thus, the mandible, including the mandibular condyle, receives substantial mechanical stimulation during the growth period and grows both vertically and horizontally. In clinical orthodontics, lateral cephalograms are regularly taken throughout treatment. Therefore, the development of ICR should be suspected when a decrease in mandibular ramus height and mandibular clockwise rotation are markedly apparent even though a patient is in a growth period [5]. This implies that orthodontists are likely to be the first to detect ICR and can contribute to its early detection.

If age-related skeletal changes are incomprehensive, findings from clinical and intraoral examination and radiography are key to the diagnosis of ICR [4,5,8]. Through clinical examination, we can obtain information about age, gender, previous orthodontic treatment, history of maxillofacial trauma, history of oral contraceptive use, the presence of TMD symptoms, and parafunctional and postural habits. As for intraoral findings, the presence of tooth abrasion on the upper and lower incisal edges and of dental compensations are useful for confirming whether the changes are due to rapid mandibular condylar resorption if an anterior open bite is observed. Unquestionably, the most important findings for a definitive diagnosis of ICR are those obtained via radiography. Panoramic radiographs can be used to determine the presence or absence of changes in mandibular condylar morphology, but three-dimensional CT is more suitable for observing the pathological details of the mandibular condyle. If destructive changes associated with mandibular condylar resorption, along with other findings, are observed, ICR can be diagnosed.

When patients present the above findings before or during treatment and are diagnosed as suffering from ICR based on radiographic findings, all treatment procedures should be interrupted at once. If necessary, splint and/or physical therapy may be applied to relieve pain, but no effective modalities for modulating the progression of ICR have been developed, and no basic studies on the development of new effective modalities for ICR have been reported. In order to determine when to initiate or resume treatment, it is essential to confirm via CT or other radiological examination that the progression of mandibular condylar resorption has ceased.

## 4. Final Remarks

ICR is still an unknown disease both in terms of its pathogenesis and risk factors, and no diagnostic criteria allowing for the early detection of this disease have been established. Little information is available on the best treatment to apply after ICR has developed. On the other hand, osteoarthritis of the knee and hip joints has been shown to be a multifactorial genetic and lifestyle-related disease caused by the interaction of genetic and environmental factors, and the genetic factors for osteoarthritis, or disease-related genes, include Asporin, Growth Differentiation Factor 5 (GDF5), and double von Willebrand factor A domains (DVWA) [19,20,21]. These disease-related genes have been found to share certain common polymorphisms in the gene regions and to correlate strongly with osteoarthritis. For example, asporin is an extracellular substrate protein belonging to the SLRP family, and a repeat polymorphism (D14) of aspartic acid (D) at the N-terminus is thought to be associated with the development of osteoarthritis because it strongly inhibits chondrogenic differentiation by TGF-β. In addition, a single-nucleotide polymorphism (SNP) that causes amino acid substitutions in proteins made by the DVWA gene was found to be strongly correlated with the development of knee osteoarthritis. This finding is expected to result in the identification of new metabolic pathways leading to the onset of osteoarthritis and to the development of good-as-new medicine for osteoarthritis via the comprehensive identification of the genes related to these pathways. Therefore, the search for the causative genes of ICR is an urgent issue, and clarification of the causes will lead to early detection, prevention, and the development of treatment methods.
